# Surgical Management Outcome of Intestinal Obstruction and Its Associated Factors at University of Gondar Comprehensive Specialized Hospital, Northwest Ethiopia, 2018

**DOI:** 10.1155/2019/6417240

**Published:** 2019-07-28

**Authors:** Tesfamichael G. Mariam, Addisu Taye Abate, Mehammed Adem Getnet

**Affiliations:** School of Nursing, College of Medicine and Health Sciences, University of Gondar, Gondar, Ethiopia

## Abstract

**Background:**

Intestinal obstruction (IO) is one of the most common acute abdominal disorders that often requires emergency surgical management in the hospital setting. However, the surgical management sometimes ends with unfavorable outcomes characterized by fatal and nonfatal postoperative complications.

**Aim:**

The aim of this study was to analyze the surgical management outcome of IO and its associated factors among surgically treated patients for intestinal obstruction at the University of Gondar Comprehensive Specialized Hospital (UGCSH), Ethiopia, 2018.

**Methods:**

An institution-based cross-sectional study was conducted among patients who were surgically treated for IO during the last 3 years at the UGCSH. The patient participants were selected using a systematic random sampling technique. A structured research tool was used to collect all the necessary data from the patients' medical records. The data were analyzed by using SPSS version 21. Frequencies with percentages were used to describe the surgical management outcome of IO. The binary logistic regression model was used to explore the determinant factors associated with the surgical management outcome of IO. Factors at *P* < 0.05 were declared statically significant.

**Results:**

227 patient participants were included and finally analyzed in this study. From these, 83.3% patients have favorable surgical management outcomes of IO, whereas the rest 16.7% patients have unfavorable outcomes. Of 38 patients with unfavorable outcome, the most common postoperative complication occurred was surgical site infection (36.8%), followed by pneumonia (23.6%) and septic shock (21.0%) among other complications. A total of 10 postoperative deaths were also documented as unfavorable surgical management outcomes of IO. Of the determinant factors analyzed in this study, only three factors, duration of illness before surgery, length of hospital stay after surgery, and comorbidity, were independently significantly associated with the surgical management outcome of IO.

**Conclusions:**

In this study, the majority of patients had favorable surgical management outcomes of IO, and the proportion of patients with unfavorable outcomes was however considerable. Thus, designing a strategy addressing the significantly associated determining factors could be helpful to further increase the likelihood of favorable surgical management outcomes of IO.

## 1. Introduction

Intestinal obstruction (IO) is a potentially risky surgical emergency associated with high morbidity and mortality rates in both developed and developing world [1, 2]. It also causes significant surgical side effects in hospital admissions and adversely affects the life of millions of people, cutting across all age groups, with considerable direct and indirect economic impacts on the healthcare system and the affected patients [3].

The incidence of IO is recognized to be high in India, Iran, Afghanistan, and certain African countries including Ethiopia. It has been the leading cause of acute abdominal disorders in Africa [4–7]. According to different studies, IO roughly accounts for about 49–60% of all surgically treated acute abdominal disorder cases in Ethiopia [7–10].

Based on the anatomical location, IO is mainly classified as small bowel obstruction (SBO) and large bowel obstruction (LBO) [2]; it can also be either mechanical or functional on the basis of the underlying pathophysiology of obstruction [11]. The etiology of IO has been varied with SBO caused by adhesions, strangulated hernia, malignancy, and volvulus [2]. The causes of IO also vary in different populations and areas. The most frequent causes of IO in the developing region are likely hernia and volvulus, whereas adhesions are most frequent in the developed region. However, there is some change about these established patterns in Africa [12–15].

Regardless of its underlying causes, a surgery for IO sometimes led to a variety of postoperative complications such as incisional site infections, wound dehiscence, pneumonia, and sepsis which are not rare especially after an emergency surgery for IO and even death as the poor outcome of that surgical management [16–18]. This outcome of surgical management can be influenced by several patient-related and clinical-related factors [14, 19]. Little is known in Ethiopia about this issue, and there are no data available on our particular study area; therefore, the aim of the present study was to assess the surgical management outcome of IO and its associated factors at a tertiary teaching hospital in northwest Ethiopia.

## 2. Methods

### 2.1. Study Design and Period

A cross-sectional study was conducted by reviewing the past three-year secondary data on patients' medical records available at the University of Gondar Comprehensive Specialized Hospital (UGCSH). The data were collected within the study period from March 30 to April 30, 2018.

### 2.2. Study Area and Setting

This study was conducted at the UGCSH, which is located in Gondar city, northwestern Ethiopia. Gondar is the capital of north Gondar zone of the Amhara region of Ethiopia. The city is one of the famous historical and cultural sites of Ethiopia, located 738 km away from the capital Addis Ababa and 180 km from the regional capital Bahir Dar.

The hospital, currently renamed as “UGCSH”, a teaching hospital under the University of Gondar's College of Medicine and Health Sciences, was established in 1954. It remains the only tertiary heathcare facility available in Gondar town. It provides the outpatient and inpatient services for more than five million people per three-tire healthcare system of Ethiopia.

The surgical department is one of the actively serving departments giving emergency and elective surgery services in the hospital. There are more than 4 operation rooms, and about 120 beds are currently available in different units or wards under the surgical department of the UGCSH.

### 2.3. Source and Study Population

The source population comprised all patients who received surgical management for IO at the UGCSH, and those who fulfilled the inclusion criteria became the study population.

#### 2.3.1. Inclusion Criteria

Patients with any age group who got surgical management of IO at the UGCSH during the last three years, from March 1, 2015, to April 30, 2018, were included in the sampling frame of this study for random selection of the required sample.

#### 2.3.2. Exclusion Criteria

Sampled patients who identified with incomplete or lost records for the major variables were excluded later from the analysis.

### 2.4. Sample Size

The sample size was initially calculated using the single population proportion formula (i.e., *n*_*i*_=*z*_*α*/2_^2^ *∗*  *p*(1 − *p*)/*d*^2^) with considering the following assumptions: *z* = 1.96, *p*=75.4% from a similar study that was conducted in Adama hospital in southeastern Ethiopia [20], and *d* = 5%. Since *N* was 743, the required minimum sample size was then adjusted using Yamane's correction formula (i.e., *n*_*f*_ = *n*_*i*_/(1 + *n*_*i*_/*N*)). Finally, by adding 10% potential nonparticipatory rate of the study, the final sample size (*n*) was 227 patients.

Here, *n*_*i*_ is the sample size before adjustment for finite population, *z* is the value of standard normal distribution curve at the 95% level of confidence, *p* is the proportion of population with favorable surgical management of IO, *d* is the margin of error, *N* is the population size, *n*_*f*_ is the sample size after adjustment for finite population, and *n* is the sample size (final).

### 2.5. Sample Selection

The systematic random sampling technique was used to select the study participants. For this purpose, all patients who had a surgery done for IO within the last three years at the UGCSH were listed by using MRN as a sampling frame. Then, considering *N* = 743, *n* = 227, and *k* = *N*/*n* ≈ 3, we randomly select the first sample as starting point, and then each sample was systematically selected for every 3 patients until *n* is reached. Here, *N* is the population size, *n* is the sample size, and *k* is the sampling interval. If any selected patient is recognized with missed or incomplete data for the major variables, they were replaced by the next sample from the sampling frame.

### 2.6. Data Collection and Variables

A structured instrument was developed in English language based on the prior literature [17, 20–22], which was used to extract all relevant data from the patients' medical records about the dependent and independent variables of this study.

#### 2.6.1. Dependent Variable

Surgical management outcome of IO.

#### 2.6.2. Independent Variables

The independent variables were as follows: *sociodemographic characteristics* (age, sex, residence, marital status, religion, and occupation), *lifestyle characteristics* (history of tobacco use, alcohol use, illicit drug use, and physical activity), *preoperative clinical characteristics* (presenting symptoms, duration of illness—from the onset of IO symptoms up to the surgery done for IO, preoperative diagnosis, preoperative cares received, comorbidity, and previous abdominal surgery), *intra- and postoperative clinical characteristics* (intraoperative diagnosis, type of intraoperative surgical procedure done, postoperative antibiotics received, and length of hospital stay—after the surgery done until discharge from the hospital inpatient service).

### 2.7. Operational Definitions

Surgical management outcome of IO, the condition of the patient after surgery done for IO that was whether she/he had “favorable” or “unfavorable” outcome according to the retrospective secondary data extracted from their medical records, was accessible at the UGCSH. The surgical management outcome of IO was defined as the dependent variable of this study, and its binary categories are described as follows:

- Unfavorable outcome: if the patient is dies or has one or more postoperative (after surgery for IO) complications like dehiscence, surgical site infection, pneumonia, and shock, as documented in the medical records.
- Favorable outcome: if the patient is discharged alive and does not have any history of postoperative (after surgery for IO) complications.

This was recognized as the surgical management outcome of each patient participant and assessed with regard to the independent variables (factors) stated in the previous subsection.

All other terminologies regarding our study variables were directly used based on the collected data extracted from the medical information record system as already available in the UGCSH study setting.

### 2.8. Data Processing and Analysis

All collected data were checked-rechecked by the investigators, then coded and entered into Epi Info version 7, and then exported into SPSS version 21 for analysis. Frequencies with percentages, means with standard deviations (SD), and/or medians with ranges were computed as appropriate to describe the entire variables of the study assessed. A binary logistic regression model was used to select the independent variables (factors) associated with the surgical management outcome of IO as the binary dependent variable. All factors with a *P* value <0.02 in the bivariate binary logistic regression analysis were considered as a candidate to be entered into the multivariate binary logistic regression analysis, in which statistical significance was based on a *P* value <0.05. For this purpose, adjusted odds ratios (AOR) with 95% confidence interval (95% CI) were calculated as a measure of the strength of the association.

### 2.9. Ethical Considerations

Ethical clearance was obtained from the Ethical Review Committee, University of Gondar College of Medicine and Health Sciences. In addition, permission letter was received from the medical director of the University of Gondar Comprehensive Specialized Hospital. All individual patients' information taken during the data collection was strictly confidential. This study has neither harm nor benefit for the participant patients.

## 3. Results

### 3.1. Sociodemographic Characteristics

A total of 227 patients who had a history of surgery for IO at the UGCSH were included and finally analyzed in this study. Of these 227 patients, 89 (39.2%) were within 5–40 years, the largest age group (Table 1). The minimum age of the patients was 3 days and the maximum was 85 years, with mean = 37.21 years, median = 38 years, and SD = 21.44. The majority (72.2%) of patients were males (Table 1), with a male-to-female ratio of 2.6 : 1. Among the total patients, 70.9% of them were rural dwellers, 66.1% married, 95.2% orthodox, and 44.5% farmers (Table 1).

### 3.2. Lifestyle Characteristics

This study has shown that 5.7% of patients had a history of ever tobacco use, 37.0% ever alcohol use, and 7.9% regular physical activity, and a history of illicit drug use is unknown for 97.8% of all patients from their medical records (Table 2).

### 3.3. Preoperative Clinical Characteristics

The findings show abdominal pain (89%), vomiting (88.1%), abdominal distension (79.7%), and failure to pass abdominal contents, such as feces and/or flatus, (76.2%) are the leading clinical symptoms among patients presenting with IO at the healthcare facility (Table 3).

The most common specific preoperative clinical diagnosis of the patients was simple SBO (65%), followed by simple LBO (18.9%) and gangrenous SBO (11.5%). Others including gangrenous LBO and incarcerated hernia are the less common preoperative clinical diagnoses identified among those patients presenting with IO at the hospital facility (Table 3).

Regarding the duration of illness, 151 (66.5%) cases are presented longer than 24 hours after the onset of IO symptoms until undergoing operation (Table 3). The duration however ranges from 2 to 120 hours among them. This study also shows 30 (13.2%) patients had a previous history of abdominal surgery, and 5.7% of all IO cases had at least one diagnosed comorbid condition of cardiovascular diseases, lung diseases, diabetes mellitus, or other chronic disorders as documented in their medical records at the UGCSH (Table 3).

Concerning the key elements of preoperative care assessed in this study, IV fluid resuscitation was given for all (100%) patients; NG tube was inserted for 123 (54.2%) patients; and preoperative prophylactic antibiotics was initiated generally for 180 (79.3%) patients (Table 3), with a combination of ceftriaxone and metronidazole (35.7%), with ceftriaxone alone (37.9%), and with ampicillin (5.7%), whereas the rest 47 (20.7%) of all patients did not received any prophylactic antibiotics before their operation for IO management.

### 3.4. Intra- and Postoperative Clinical Characteristics

Gangrenous sigmoid volvulus (GSV) was the leading specific intraoperative clinical diagnosis of IO, followed by simple small bowel volvulus (SSBV), gangrenous small bowel volvulus (GSBV), adhesion and band, intussusception, and simple sigmoid volvulus (SBV) among others (Table 4).

The commonest specific type of intraoperative procedure done, after a general laparotomy, to treat the patients with IO was resection and anastomosis. Postoperative antibiotics were initiated for the majority of patients (Table 4).

Regarding the length of hospital stay, 36.1% patients stayed in the hospital for >8 days after their surgery for IO (Table 4). The mean, median, and SD of hospital stay in days were founded to be 9.07, 7, and 7.32, respectively, with the minimum of 1 day and the maximum of 60 days.

### 3.5. Surgical Management Outcome

This study shows 189 (83.3%) of 227 patients have favorable surgical management outcomes of IO which was defined as the absence of all types of postoperative complications, whereas the rest 38 (16.7%) patients have unfavorable outcomes which was defined as the presence of one or more types of postoperative complications.  Table 5 provides the types of postoperative complications documented from 38 patients all who had unfavorable surgical management outcome of IO. Beside this, 19 cases developed a complication within the first four days of the postoperative period, 9 cases developed a complication within four to eight days, and the other 10 cases developed a complication after eight days of their postoperative period.

Furthermore, the overall success rate of the surgery is 95.6%, with 217 patients discharged on improvement, although 10 (4.4%) inpatient postoperative deaths were documented, among a total of 227 analyzed cases who were engaged for the surgical management of IO at a tertiary healthcare facility in northwestern Ethiopia.

### 3.6. Factors Associated

From the bivariate binary logistic regression analysis, factors including duration of illness, comorbidity, preoperative diagnosis, type of intraoperative procedure done, and length of hospital stay were associated with the surgical management outcome of IO (at *P* < 0.02). Subsequently, all these factors were entered into the multivariable binary logistic regression model.

In the multivariable analysis, only three factors such as duration of illness, comorbidity, and length of hospital stay were significantly associated with the surgical management outcome of IO (at *P* < 0.05), while all other factors entered are becoming insignificant.

The patients seeking healthcare for IO within 24 hours of illness were about eleven times (AOR = 11.35; 95% CI: 1.88–19.54; *P*=0.009) more likely to have a favorable outcome than those seeking healthcare after 24 hours of illness.

The patients presenting with a comorbid disease are ninety-five percent (AOR = 0.05; 95% CI: 0.01–0.33; *P*=0.002) less likely to have a favorable outcome on the surgical management of IO when compared to those without any comorbid disease.

The patients those who stayed in the hospital for ≤8 days after surgery were about three times (AOR = 3.11; 95% CI: 1.11–8.70; *P*=0.030) more likely to have favorable outcome than those who stayed in the hospital for >8 days after surgery (Table 6).

## 4. Discussion

### 4.1. Surgical Management Outcome

The aim of the present study was to analyze the surgical management outcome of intestinal obstruction and its associated factors at the University of Gondar Comprehensive Specialized Hospital. The analyzed data showed that 16.7% of all cases have unfavorable surgical management outcomes of IO, which was characterized by the presence of the recorded postoperative complications or death at the healthcare facility. The finding on this unfavorable outcome rate is in line with a study conducted in Debre Berhan Referral Hospital in northeastern Ethiopia [21], but it is lower than the studies from eastern and southeastern Ethiopia, such as 32.8% in Gelemso General Hospital [22] and 24.6% in Adama Hospital [20], and also lower than the findings from other countries, such as 24.6% in India [23] and 24.2% in Uganda [7]. The possible reason for the difference might be due to variation in the distribution of the clinical and sociodemographic characteristics of the study participants, the knowledge and skill of the health professionals regarding the diagnosis and management of IO, the hospital internal setups itself, and the overall infrastructures of the study area, as well as may depend on the operational definitions used between the literature.

### 4.2. Associated Factors

Our study revealed that duration of illness before surgery, length of hospital stay after surgery, and comorbidity were factors significantly associated with the surgical management outcome of IO. The patients who presented in the hospital within 24 hours of duration of illness were about 11 times more likely to have favorable surgical management outcome compared with those who presented longer than 24 hours after the onset of IO symptoms before surgery. This finding is supported by the research studies conducted in southwestern Ethiopia and in Rwanda [14, 20] but not supported by a research study done in Turkey [24]. The possible reason for the deliance of patients to attending a hospital might be due to lack of awareness about the symptoms of IO, inadequate infrastructures, and transport accessibility especially for those patients who reside in rural areas, their distance from the primary and\or tertiary healthcare facilities, and poor referral system between the levels of health facilities.

This study also shows that patients who stayed in the hospital for shorter than 8 days after surgery were about 3 times more likely to have favorable outcomes when compared with those who stayed for longer than or equal to 8 days after the surgery. It is consistent with other studies conducted in Ethiopia [8, 22] and in Uganda [24]. The short length of hospital stay may decrease the chance of patients to acquire nosocomial infections, such as hospital-acquired pneumonia.

Another significantly associated factor in this study is comorbidity. If a patient had one or more comorbid diseases, it is about 95% less likely that the patient would have a favorable surgical management outcome of IO when compared to those without any comorbid disease. This finding is congruent with a study conducted in Turkey [1]. The comorbid conditions like diabetes may decrease the wound-healing process and increase the risk of postoperative complications' occurrence as an unfavorable surgical management outcome of IO including incisional site infection and wound dehiscence.

## 5. Conclusions

This study provided insight into the surgical management outcome and its factors associated among patients with intestinal obstruction at a tertiary teaching hospital in northwestern Ethiopia. The majority of patients had favorable surgical management outcomes of IO, and the proportion of patients with unfavorable outcomes was however somewhat considerable. Determinant factors including duration of illness before surgery, length of hospital stay after surgery, and comorbidity were significantly associated with the surgical management outcome of IO. Therefore, designing a strategy addressing these factors would be helpful to further increase the likelihood of favorable surgical management outcome for the patients attending hospital with IO.

## Figures and Tables

**Table 1:** Sociodemographic characteristics of IO patients (*N* = 227) at UGCSH, Ethiopia.

Variable	Category	Frequency	%
Age (years)	<5	26	11.5
	5–14	15	6.6
	15–40	89	39.2
	41–60	59	26.0
	>60	38	16.7

Sex	Male	164	72.2
	Female	63	27.8

Residence	Rural	161	70.9
	Urban	66	29.1

Marital status	Not eligible	37	16.3
	Single	34	15.0
	Married	150	66.1
	Divorced	6	2.6

Religion	Orthodox	216	95.2
	Muslim	11	4.8

Occupation	Farmer	101	44.5
	Merchant	11	4.8
	Housewife	33	14.5
	Student	43	18.9
	Government employee	9	4.0
	Others	30	13.2

**Table 2:** Lifestyle characteristics of IO patients (*N* = 227) at UGCSH, Ethiopia.

Variable	Category	Frequency	%
Tobacco use	Ever	13	5.7
	Never	45	19.8
	Unknown^*∗*^	169	74.5

Alcohol use	Ever	84	37.0
	Never	27	11.9
	Unknown^*∗*^	116	51.1

Illicit drug use	Yes	—	—
	No	5	2.2
	Unknown^*∗*^	222	97.8

Regular physical activity	Yes	18	7.9
	No	53	23.4
	Unknown^*∗*^	156	68.7

^*∗*^There is no information identified from the source about that context, after extensive retrieval of the medical records.

**Table 3:** Preoperative clinical characteristics of IO patients (*N* = 227) at UGCSH, Ethiopia.

Variables^*∗*^	Category	Frequency	%
Presenting symptoms	Abdominal pain	202	89.0
	Abdominal distension	181	79.9
	Vomiting	200	88.1
	Constipation	11	4.8
	Failure to pass flatus/feces	173	76.2
	Diarrhea	10	4.4
	Fever	3	1.3

Preoperative diagnosis	Simple SBO	119	52.4
	Simple LBO	43	18.9
	Gangrenous SBO	26	11.5
	Gangrenous LBO	32	14.1
	Incarcerated hernia	7	3.1

Preoperative care	Antibiotics initiated	180	79.3
	NG tube inserted	123	54.2
	IV fluid resuscitation	227	100

Duration of illness (hours)	>24	151	66.5
	≤24	76	33.5
Comorbidity	Yes	13	5.7
	(i) Cardiovascular disease	2	0.9
	(ii) Lung disease	2	0.9
	(iii) Diabetes mellitus	4	1.7
	(iv) Others	5	2.2
	No	214	94.3

Previous abdominal surgery	Yes	30	13.2
	No	197	86.8

^*∗*^Some variables have possibly multiple responses.

**Table 4:** Intra- and postoperative clinical characteristics of IO patients (*N* = 227) at UGCSH, Ethiopia.

Variables	Category	Frequency	%
Intraoperative diagnosis	SSV	21	9.3
	GSV	53	23.3
	SSBV	42	18.5
	GSBV	38	16.7
	Incarcerated hernia	6	2.6
	Strangulated hernia	7	3.1
	Adhesion and band	30	13.2
	Intussusception	22	9.7
	Tumor and carcinoma	8	3.5

Intraoperative procedure	Untwisting the volvulus	50	22.0
	Resection and anastomosis	99	43.6
	Hartman's colostomy	22	9.7
	Herniorrhaphy	13	5.7
	Adhesiolysis and band release	29	12.8
	Manual reduction	14	6.2

Postoperative antibiotics	Not initiated at all	44	19.4
	Initiated ≤5 days after surgery	120	52.9
	Initiated >5 days after surgery	63	27.7

Length of hospital stay (days)	≤8	145	63.9
	>8	82	36.1

**Table 5:** Types of postoperative complications documented from patients who had unfavorable surgical management outcomes of IO (*N* = 38) at UGCSH, Ethiopia.

Postoperative complication	Category	Frequency	%
Hematoma or serosa	Yes	1	2.7
	No	37	97.3

Superficial incisional SSI	Yes	4	10.5
	No	34	89.5

Deep incisional SSI	Yes	5	13.0
	No	33	87.0

Anastomotic leak (organ SSI)	Yes	5	13.0
	No	33	87.0

Facial dehiscence	Yes	6	15.7
	No	32	84.3

Pneumonia	Yes	9	23.6
	No	29	76.4

Septic shock	Yes	8	21.0
	No	30	79.0

Hypokalemia	Yes	1	2.7
	No	37	97.3

Respiratory failure	Yes	4	10.5
	No	34	89.5

Sudden cardiac arrest	Yes	1	2.7
	No	37	97.3

Hypnotic encephalopathy	Yes	1	2.7
	No	37	97.3

Postoperative death occurred	Yes	10	26.3
	No	28	73.7

**Table 6:** Factors associated with the surgical management outcome of IO at UGCSH, Ethiopia.

Variables	Category	Outcome^*∗*^		AOR (95% CI)	*P*
		Fav. (*n*)	Unfav. (*n*)		
Duration of illness	≤24 hours	74	2	11.35 (1.88–19.54)	0.009
	>24 hours	115	36	1	

Comorbidity	Yes	5	7	0.05 (0.01–0.33)	0.002
	No	184	31	1	

Preoperative diagnosis	Simple SBO	106	13	1	
	Simple LBO	36	7	0.31 (0.04–2.65)	0.286
	Gangrenous SBO	12	14	0.39 (0.08–1.80)	0.225
	Gangrenous LBO	28	4	0.65 (0.08–5.51)	0.692

Intraoperative diagnosis	SSV	20	1	2.70 (0.06–11.27)	0.604
	GSV	46	7	0.71 (0.02–29.44)	0.855
	GSBV	23	15	0.15 (0.01–4.34)	0.270
	Adhesion and band	25	5	0.04 (0.01–2.05)	0.107
	Intussusception	17	5	0.13 (0.01–1.02)	0.051
	Tumor and carcinoma	4	4	0.17 (0.01–7.86)	0.364
	SSBV	41	1	1	

Intraoperative procedure	Untwisting the volvulus	48	2	1	
	Resection and anastomosis	70	29	0.86 (0.06–12.69)	0.916
	Hartman's colostomy	20	2	5.88 (0.21–16.95)	0.301
	Adhesiolysis	26	3	8.94 (0.19–43.18)	0.265
	Manual reduction	12	2	7.36 (0.16–26.01)	0.323

Length of hospital stay	≤8 days	130	15	3.11 (1.11–8.70)	0.030
	>8 days	59	23	1	

^*∗*^Surgical management outcome of intestinal obstruction. Fav., favorable. Unfav., unfavorable. *n*, count. AOR, adjusted odds ratio. CI, confidence interval. 1, reference level. *P*, *P* value (level of significance).

## Data Availability

The data used to support the findings of this study are available from the corresponding author upon request.

## Abbreviations

AOR: Adjusted odds ratio
CI: Confidence interval
Epi Info: Statistical package for epidemiological information analysis
GSBV: Gangrenous small bowel volvulus
GSV: Gangrenous sigmoid volvulus
IO: Intestinal obstruction
IV: Intravascular
LBO: Large bowel obstruction
MRN: Medical record number
NG: Nasogastric
SBO: Small bowel obstruction
SD: Standard deviation
SPSS: Statistical Package for Social Science
SSBV: Simple small bowel volvulus
SSI: Surgical site infection
SSV: Simple sigmoid volvulus
UGCSH: University of Gondar Comprehensive Specialized Hospital.
